# Phosphatidylinositol 3-kinase (PI3K) inhibitors as cancer therapeutics

**DOI:** 10.1186/1756-8722-6-88

**Published:** 2013-11-22

**Authors:** Akintunde Akinleye, Parthu Avvaru, Muhammad Furqan, Yongping Song, Delong Liu

**Affiliations:** 1 Division of Hematology/Oncology, Department of Medicine, New York Medical College and Westchester Medical Center, Valhalla, NY 10595, USA; 2 Henan Cancer Hospital, Zhengzhou University, Zhengzhou, China

## Abstract

Phosphatidylinositol 3-kinases (PI3Ks) are lipid kinases that regulate diverse cellular processes including proliferation, adhesion, survival, and motility. Dysregulated PI3K pathway signaling occurs in one-third of human tumors. Aberrantly activated PI3K signaling also confers sensitivity and resistance to conventional therapies. PI3K has been recognized as an attractive molecular target for novel anti-cancer molecules. In the last few years, several classes of potent and selective small molecule PI3K inhibitors have been developed, and at least fifteen compounds have progressed into clinical trials as new anticancer drugs. Among these, idelalisib has advanced to phase III trials in patients with advanced indolent non-Hodgkin’s lymphoma and mantle cell lymphoma. In this review, we summarized the major molecules of PI3K signaling pathway, and discussed the preclinical models and clinical trials of potent small-molecule PI3K inhibitors.

## Introduction

**Figure 1:**
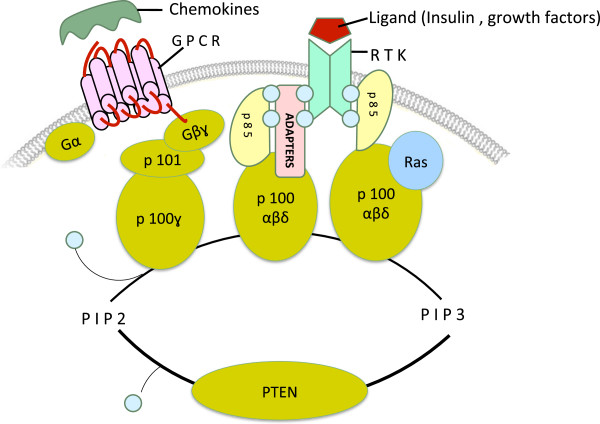
**The class I PI3K family.** Class I PI3Ks are heterodimeric proteins and comprised of a catalytic p110 subunit complexed with a regulatory p85 or p101 subunit. The catalytic p110 subunit exists in four isoforms (α, β, δ, and γ), whereas the regulatory p85 subunit in three isoforms – p85, p55, and p50. Their corresponding upstream receptors and adaptor proteins are also indicated. RTK: receptor tyrosine kinase; GPCR: G-protein coupled receptors.

Phosphatidylinositol 3-kinases (PI3Ks) are lipid kinases that play central role in regulation of cell cycle, apoptosis, DNA repair, senescence, angiogenesis, cellular metabolism, and motility [1]. They act as intermediate signaling molecules and are most well known for their roles in the PI3K/AKT/mTOR signaling pathway [2,3]. PI3Ks transmit signals from the cell surface to the cytoplasm by generating second messengers – phosphorylated phosphatidylinositols – which in turn activate multiple effector kinase pathways, including BTK, AKT, PKC, NF-kappa-B, and JNK/SAPK pathways, and ultimately result in survival and growth of normal cells [1-5] (Figure 1). Although the activity of PI3Ks is tightly regulated in normal cells by internal signals such as PTEN (phosphatase and tensin homolog deleted from chromosome 10), it has been recognized that deregulation of the PI3K signaling pathway is associated with development in one-third of human cancers [6-9]. Aberrantly activated PI3K pathway promotes carcinogenesis and tumor angiogenesis [3,10-12]. For example, approximately 30% of breast cancers demonstrated activating missense mutations of *PIK3CA*, the gene encoding the catalytic p110α subunit of class I PI3K, and the mutated gene provides cells with a growth advantage and promotes tumorigenesis [13]. In addition, dysregulated PI3K pathway signaling has been implicated in conferring resistance to conventional therapies including biologics, hormonal therapy, tyrosine kinase inhibitors, radiation, and cytotoxics in breast cancer, glioblastoma, and non-small cell lung cancer [2,14]. Other genetic aberrations that drive the PI3K pathway in cancer include gene amplification of PI3Ks, loss of the regulatory activity of PTEN, and activating mutations of receptor tyrosine kinases (RTKs) such as EGFR and HER2 [13,15-18]. With this background, PI3K has become recognized within the last decade as a viable target for novel anti-cancer therapy. Successful drug design has yielded several classes of potent, selective, and efficacious small molecule PI3K inhibitors that are currently at different stages of development. Idelalisib, which represents the first-in-class oral PI3K p110-δ inhibitor, was efficacious with an acceptable safety and tolerability profile in early phase studies, and has progressed into phase III clinical trials in patients with advanced indolent non-Hodgkin’s lymphoma (iNHL), chronic lymphocytic leukemia (CLL) and mantle cell lymphoma (MCL) [19-23]. In this comprehensive review, we provide an overview of the PI3K signaling pathway in tumorigenesis and highlight recent advances in the design of small-molecule inhibitors of PI3K as novel anti-cancer therapies. In addition, this review discusses the most recent preclinical and clinical studies of inhibitors targeting the different isoforms of the PI3K enzymes in the treatment of hematological and solid malignancies.

### PI3K signaling pathway in health and tumorigenesis

**Figure 2:**
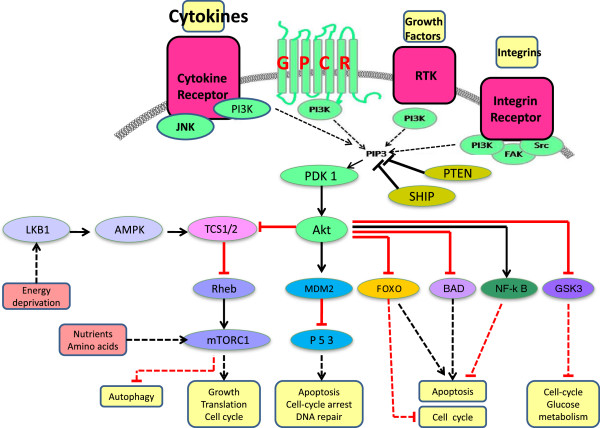
**Schematic representation of the PI3K signaling pathway.** Four major extracellular signals, growth factors, cytokines, hormones/chemokines, and integrins, activate PI3K, which transmit the signals through appropriate pathways to control diverse cellular processes, including cell cycle, apoptosis, DNA repair, senescence, angiogenesis, cellular metabolism, autophagy, and motility. The multiple effector kinase pathways activated by PI3K are highlighted in the figure.

PI3Ks represent a family of lipid kinases that lie upstream of complex, intricate, interconnected intracellular signaling networks [1] (Figure 2). They transduce signals from transmembrane receptors such as RTKs and G-protein coupled receptors (GPCRs) to the cytoplasm – through production of phosphorylated lipids – to regulate key cellular processes including proliferation, differentiation, senescence, motility, and survival [13].

**Figure 3:**
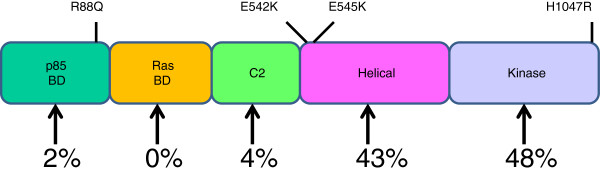
**The structural organization of p110-α enzyme.** The catalytic subunit (p110-α) of PI3Ks possesses a central region flanked by the N- and C-terminus of varying lengths with distinctive modular organization. The N-terminus of p110-α enzyme harbors the p85-binding domain (PI3K-ABD) and the Ras-binding domain (PI3K-RBD) which mediates interaction with the regulatory p85 and the Ras-GTPases respectively. The central region is composed of the C2 PI3K-type and PIK helical domains, whereas the C-terminus houses the enzymatic apparatus (PI3K/PI4K kinase domain). Common cancer-associated mutations within each domain of the enzyme is indicated.

PI3Ks are enzymes of approximately 200–300 kDa in molecular weight (Figure 3). In human, three distinct classes of PI3Ks (I – III) have been identified (Table 1). They differ on basis of their structural characteristics, substrate specificities, and nature of lipid end-products. Class I PI3Ks are heterodimers and further divided into 2 subfamilies, IA and IB. Class IA PI3Ks are the most studied and frequently implicated in cancer [24,25]. Structurally, class IA PI3Ks comprise of catalytic p110 complexed with regulatory p85 subunits. The catalytic p110 isoforms (α, β, and δ) are encoded by the genes *PIK3CA*, *PIK3CB*, and *PIK3CD* respectively, whereas the regulatory p85 subunit– p85, p55, and p50 isoforms – are encoded by *PIK3R1, PIK3R2,* and *PIK3R3* genes, respectively [26,27]. Class IB PI3Ks also consist of catalytic p110γ and regulatory p101, and p84/p87PIKAP subunits [27]. Likewise, class III PI3Ks are heterodimeric proteins having a catalytic (hVps34) subunit associated with regulatory (p150) subunit. The regulatory subunit subserves 2 functions [28]. Upon receptor activation, it recruits the catalytic subunit to tyrosine phosphorylated proteins (RTKs, adaptors) at the plasma membrane where the catalytic subunit phosphorylates its lipid substrates [27]. In addition, the enzymatic activity of the catalytic subunit is constitutively inhibited by the regulatory subunit in quiescent cells [28]. Class II PI3K enzymes also exist in 3 isoforms (PI3KC2α, PI3KC2β and PI3KC2γ). However, these are monomers with high molecular weight, lack regulatory subunits, and possess single catalytic unit that directly interacts with phosphorylated adapter proteins [26,29]. The catalytic units of PI3Ks possess an N-terminal sequence, a central region, and a C-terminus; however the modular organizations are distinctive. The N-terminus of class IA p110 (α, β, and δ) enzymes harbors the p85- binding domain (PI3K-ABD), which constitutively interacts with the SH2 domain of the regulatory subunit, and also houses the Ras-binding domain (PI3K-RBD) which mediates interaction with Ras-GTPases. The central region is comprised of the C2 PI3K-type and PIK helical domains, whereas the C-terminus contains the catalytic apparatus (PI3K/PI4K kinase domain). The PI3K-RBD domain is the most divergent region of the class IA enzymes [25]. The class IB enzyme, p110γ, is similar in structural organization to the class IA p110 proteins but also contains a putative N-terminus PH domain [30]. In class II enzymes, however, the central region is made-up of four domains (PI3K-RBD, C2 PI3K-type, PIK helical, PI3K/PI4K kinase), and the C-terminal sequence composed of the C2, and PX domains. The N-termini of class II PI3Ks are more distantly related. This region contains the binding site for GRB2 (Growth factor receptor-bound protein 2), an adapter protein that often complexes with SOS and Ras-GTPases, and facilitates recruitment and activation of PI3KC2α and PI3KC2β by activated growth factor receptors [31]. In addition, the N-terminal sequence of PI3KC2α also serves as major binding site for clathrin trimers and thereby independently modulating clathrin distribution and function [32,33]. Class III catalytic enzyme, hVps34, is characterized by an N-terminal C2 PI3K-type domain, a centrally located PIK helical domain, and a C-terminus PI3K/PI4K kinase domain [34].

**Table 1:** Phosphatidylinositol-3 kinase genes and proteins

**Class**	**Gene**	**Chromosomal location**	**Protein**	**Mass (kDa)**	**Sequence length (AA)**
Class I					
IA	*PIK3CA*	3q26.3	p110-α	124.28	1068
	*PIK3CB*	3q22.3	p110-β	122.76	1070
	*PIK3CD*	1p36.2	p110-δ	119.48	1044
	*PIK3R1*	5q13.1	p85-α	83.60	724
	*PIK3R2*	19p13.1	p85-β	81.55	728
	*PIK3R3*	1p34.1	p55-γ	54.45	461
IB					
	*PIK3CG*	7q22.3	π110-γ	126.45	1102
	*PIK3R5*	17p13.1	p101	97.35	880
	*PIK3R6*	17p13.1	p84/p87PIKAP	84.26	754
Class II					
	*PIK3C2A*	11p15.1	PIK-C2α	190.68	1686
	*PIK3C2B*	1q32.1	PIK-C2β	184.77	1634
	*PIK3C2G*	12p12.3	PIK-C2γ	165.72	1445
Class III					
	*PIK3C3*	18q12.3	hVps34	101.55	887
	*PIK3R4*	3q22.1	p150	153.10	1358

*Abbreviations*: *AA* amino acids; *kDa* kilodalton.

P110α and p100β are ubiquitously expressed in all tissues, whereas p110δ is mostly confined to hematopoietic cells, where it plays an important role in B-cell homeostasis and functioning. These enzymes integrate inputs from activated RTKs and GPCRs [25]. The p110γ, predominantly expressed by pancreas, skeletal muscles, liver and heart, mediates signaling downstream of GPCRs [30]. Class II PI3Ks are widely expressed at varying levels in all tissues, and activated by RTKs, cytokine receptors, chemokine receptors, and integrins [31,32]. Similarly, hVps34 is ubiquitously expressed, with the highest expression in skeletal muscle, and plays a key role in diverse intracellular trafficking in the cytosolic compartment of the cells [35].

PI3Ks are predominantly cytosolic, non-phosphorylated and catalytically inactive in quiescent cells except class II PI3Ks which preferentially associate with membrane fraction of cells [32]. In response to growth factor stimulation, tyrosine phosphate motifs of activated receptors recruit PI3Ks to the plasma membrane by direct interaction with the SH2 domains of the regulatory subunit [36]. This interaction also alters the conformation of the regulatory subunit, abrogates its inhibitory activity, and causes full activation of the enzymatic activity of the catalytic subunit [28]. PI3Ks can also be stimulated by activated Ras-GTPases that exist in a complex with phosphorylated adapter proteins (GRB2, SOS) [8,26,31]. These activated PI3Ks then catalyze the generation of second messengers – phosphorylated phosphatidylinositols (PI) – which in turn activate multiple downstream signaling pathways [1]. In vitro, class I PI3Ks are capable of phosphorylating PI to PI 3-phosphate; PI 4-phosphate to PI 3,4-bisphosphate; and PI 4,5-bisphosphate to PI 3,4,5-trisphosphate. However PI 4,5-bisphosphate (PIP2) is the preferred lipid substrate in vivo [27]. hVps34, the class III PI3K enzyme, mainly catalyzes the conversion of PI to PI 3-phosphate to mediate cellular trafficking processes [27,34], while class II enzymes utilize PI, PIP2, and PI 4-phosphate as substrates to generate PIP3 and PI 3,4-bisphosphate in vivo [32,37-39].

PI3K signaling regulates a wide range of cellular processes including protein synthesis, cell survival, proliferation, differentiation, senescence, motility, angiogenesis and metabolism. Upon generation of second messengers (PIP3, PI 3,4-bisphosphate), the PI3K signaling impinges on a diverse array of pleckstrin homology (PH) domain-containing intracellular signaling proteins, and indirectly triggers a cascade of events that culminates in activation of multiple effector kinase pathways, including the mTOR, ERK1/2, p38 MAPK, NF-kappa-B, and JNK/SAPK pathways [1,40,41]. These signaling proteins include serine-threonine kinases (AKT and PDK1), protein tyrosine kinases (Tec/BTK family), exchange factors for GTP-binding proteins (Grp1 and Rac exchange factors), cytoskeletal proteins, and adapter proteins (GAB-1) [4,27]. Of note, PIP_3_ binds to the PH domains of AKT and PDK1, recruits both molecules to the plasma membrane in close proximity where AKT is activated by phosphorylation at Tyr-308 by PDK1 [42-44]. PI3K-AKT signaling pathway promotes cell growth and survival by several mechanisms. Recent studies suggest that activated AKT has direct effect on the apoptosis pathway by targeting and downregulating the pro-apoptotic activity of Bcl-2 family members BAD and BAX resulting in cell survival [1]. Furthermore, PI3K-AKT signaling controls cell death and survival through NF-*kappa*-B regulation of pro- and anti-apoptotic genes [45]. AKT also signals to a few other proteins, such as mammalian target of rapamycin (mTOR) –containing protein complex mTORC1, GSK3 (glycogen synthase kinase 3), TSC (tuberous sclerosis complex), and FOXOs (the forkhead family of transcription factors), and thereby regulates cell proliferation, protein synthesis and glucose metabolism [46-48]. Besides the PI3K-AKT pathway, several other pathways, such as those of BTK/Tec kinases, have also recently been characterized [4]. The PI3K-BTK signaling plays an essential role in orderly B-cell development, proliferation and survival through recruitment and activation by CD19 [49,50]. In response to CD28 costimulation, PI3K upregulates BCL-XL expression in T-cells, and confers resistance to apoptosis during their activation [51]. In addition to its pro-survival and growth-promoting roles, the PI3K pathway is essential in endothelial cell migration during angiogenesis through VEGF-A signaling [52,53], required for lymphatic vasculature development via signaling by EGF and FGF2, and also participates in cardiomyogenesis from embryonic stem cells [54].

The lipid end-products of PI3Ks are barely detectable in unstimulated cells. The cellular levels of the second messengers are tightly regulated by the opposing action of at least three different types of phosphatases. PTEN can reduce the cellular pool of PIP3 by converting PIP3 back to inactive PIP2 through dephosphorylation at the D3 position, whereas the Src-homology 2 (SH2)-containing phosphatases (SHIP1 and SHIP2) specifically hydrolyze the D5 phosphate group of PIP3 to produce PI 3,4-bisphosphate [55]. The activity of SHIP1 and SHIP2 only partially downregulate PI3K signaling as PI 3,4-bisphosphate can also mediate PI3K-dependent responses independent of those stimulated by PIP3 [1]. Full termination of PI3K signaling is carried out by the concerted actions of inositol polyphosphate 4-phosphatase type II (INPP4B) and myotubularin, which preferentially hydrolyze PI 3,4-bisphosphate to PI 3-phosphate, and PI 3-phosphate to PI respectively [56-58].

Given its pivotal role in preventing apoptosis and stimulating proliferation in normal cells, it is not surprising that the PI3K signaling pathway is dysregulated frequently in human cancers, and exploited by tumor cells for increased proliferative potential, evasion of apoptosis, tissue invasion, and metastasis [3,27]. The PI3K signaling is aberrantly activated by at least three major mechanisms including activating mutations or amplification of catalytic subunits of PI3Ks, inactivation of the lipid phosphatase PTEN, and receptor amplification or mutations (RTKs, GPCR [12,16]. For instance, approximately 30% of breast cancers are associated with activating missense mutations of *PIK3CA*, the gene encoding the catalytic p110α subunit of class IA PI3K, which provides cells with a growth advantage and promotes tumor progression [13]. Somatic loss of PTEN activity by gene mutation, epigenetic silencing or deletion is associated with significantly greater Gleason score, poorer prognosis, and higher rate of metastasis in prostate cancer [59,60]. Increased p110 β activity due to gene amplification is frequent in human colon cancer (70%), and confers limitless growth potential [61]. Recent cancer genomic analysis showed that *PIK3R1,* the gene encoding the p85α regulatory subunit, was mutated in up to 10% of human glioblastomas [62].

PI3Ks have therefore emerged as viable targets for novel anti-cancer therapy. Successful drug design has yielded three classes of potent and selective small molecule inhibitors that have progressed from advanced preclinical testing to different stages of clinical development. Idelalisib, which represents the first-in-class oral PI3K p110-δ inhibitor, demonstrated high efficacy and a good safety profile in early phase studies. It has progressed into phase III clinical trials in patients with advanced indolent non-Hodgkin’s lymphoma (iNHL) and mantle cell lymphoma (MCL) [19-23,63].

### PI3K inhibitors in clinical development

PI3K inhibitors are divided into three classes, pan-class I, isoform-selective and dual PI3K/mTOR inhibitors, based on pharmacokinetic properties and isoform selectivity for the ATP binding site of PI3Ks [64,65] (Table 2). In the pan-class I PI3K inhibitors, wortmannin and LY294002 represent the first generation inhibitors with highly potent PI3K-inhibitory property. Notably, wortmannin and LY294002 inhibit PI3Ks activity in vitro at IC50 of 1 nM and 1.4 uM, respectively [66-68]. However, these compounds demonstrated considerable toxicities in animal studies and were not advanced to clinical evaluation because of this pharmaceutical limitation [69,70]*.* Nonetheless, at least 15 agents are in various stages of clinical development, with favorable safety, efficacy, pharmacokinetics, and pharmacodynamics profiles. GDC-0941 was first to enter clinical trials but idelalisib is now the most advanced.

**Table 2:** PI3K inhibitors in clinical trials

**Drug**	**Target(s)**	**Tumors**	**Toxicities**	**Clinical trials**	**References**
Idelalisib (CAL-101)	p110-δ	CLL/SLL, iNHL, MCL	Pyrexia, nausea, decrease appetite, fatigue	III	[76,77,80,83,86,88-90]
Buparlisib (BKM-120)	p110-α,-β, -δ,-γ	Breast, GBM, NSCLC	Rash, hyperglycemia diarrhea, anorexia	IB/II	[103-109]
GDC-0941	p110-α,-β, -δ,-γ	Breast, NSCLC, melanoma endometrial, pancreatic	Nausea, diarrhea, rash vomiting, anorexia	IB/II	[117-123]
PX-866	p110-α,-β, -δ,-γ	Ovarian, prostate, GBM NSCLC	Fatigue, diarrhea thromboembolism	II	[126-129]
GDC-0032	p110-α, -δ,-γ	Breast, NSCLC	Diarrhea, hyperglycemia fatigue, nausea, decreased appetite	I	[132]
BAY 80-6946	p110-α,-β	NHL, esophageal, sarcoma pancreatic	Alopecia, dysgeusia anemia, mucositis	I	[135-137]
IPI-145	p110-δ,-γ	CLL/SLL, iNHL, MCL	Cytopenias liver enzyme elevations	I	[138,139]
BEZ-235	p110-α,-β, -δ,-γ/mTOR	Breast, GBM	Mucositis	IB/II	[149-152]
BYL-719	p110-α	Breast, cervical, endometrial ovarian, H&N	Nausea, diarrhea hyperglycemia, vomiting	IB/II	[153-155]
BGT-226	p110-α,-β, -δ,-γ/mTOR	Solid tumors, breast	Nausea, vomiting diarrhea	I/II	[156]
PF-04691502	p110-α,-β, -δ,-γ/mTOR	Endometrial	Fatigue, nausea, vomiting decreased appetite, rash	II	[162]
GDC-0980	p110-α,-β, -δ,-γ/mTOR	Prostate	Hyperglycemia, rash mucositis	IB/II	[167,168]
GSK-2126458	p110-α,-β, -δ,-γ/mTOR	RCC, bladder	Nausea, vomiting diarrhea	I	[169,170]
PF-05212384	p110-α,-γ/mTOR	Solid tumor, CRC	Rash, mucositis transaminitis, hyperglycemia	II	[172]
XL-765	p110-α,-β, -δ,-γ/mTOR	NSCLC, gliomas	Nausea, diarrhea elevated liver enzymes	IB/II	[176-178]
XL-147	p110-α,-β, -δ,-γ	Solid tumor, GBM	Nausea, vomiting diarrhea	I/II	[180-183]

*Abbreviations*: *CLL/SLL* chronic lymphocytic leukemia/small lymphocytic leukemia; *CRC* colorectal cancer; *GBM* glioblastoma multiforme; *H&N* head and neck cancer; *iNHL* indolent non-Hodgkin’s lymphoma; *MCL* mantle cell lymphoma; *NHL* non-Hodgkin’s lymphoma; *NSCLC* non-small cell lung cancer; *RCC* renal cell cancer.

### Idelalisib (CAL-101, GS-1101)

Idelalisib (formerly CAL-101, GS-1101) is an oral, first-in-class, highly selective inhibitor of PI3K p110-δ isoform that was identified in a kinome-wide screen using purified enzymes [19,71]. A phenylquinazolin derivative, idelalisib demonstrated 240- to 2500-fold selectivity for p110δ over the other class I PI3K isoforms in cell-based assays [71], exerted far greater pro-apoptotic activity in B-ALL and CLL cell lines compared with AML cells in a dose- and time-dependent fashion [71,72], and inhibited CLL cell chemotaxis toward CXCL12 and CXCL13 [73]. The compound also suppresses survival signals provided by the microenvironment in CLL cell lines [71]. Treatment with idelalisib induces cell cycle arrest and apoptosis in Hodgkin’s lymphoma cell lines [74]. In addition, idelalisib demonstrated cytotoxicity against LB and INA-6 myeloma cell lines [75]. Importantly, idelalisib does not increase apoptosis in normal T / NK cells, nor does it block antibody-dependent cellular cytotoxicity, but the inhibitor can decrease the level of various inflammatory and anti-apoptotic cytokines from activated T cells [72]. These studies provided strong rationale for clinical trials of idelalisib as a targeted therapy for B-cell lymphoproliferative disorders.

It was reported that single agent idelalisib at doses of 50–350 mg BID demonstrated acceptable toxicity profile, positive pharmacodynamic effects, and favorable clinical activity in heavily pretreated patients with relapsed/refractory CLL, including those with adverse cytogenetics [76,77]. The final results of this phase I trial, presented at the 2013 American Society of Clinical Oncology (ASCO) meeting, showed an impressive 56% overall response rate (ORR), 17 months median progression free survival (PFS), and 18 months median duration of response (DOR) in patients treated with idelalisib alone [20]. Clearly, this study demonstrated that the activity of single-agent idelalisib in relapsed/refractory CLL is superior to current standard therapies [78,79]. Serious adverse events of pneumonia, neutropenia, thrombocytopenia, neutropenic fever, anemia, and ALT/AST elevations were observed with idelalisib treatment. A dose of 150 mg BID was brought forward for subsequent studies [20]. Idelalisib has also shown promising single-agent activity in relapsed/refractory MCL [21,80], yielding response rates similar to those previously reported for standard single-agent therapies in this setting [81,82]. Long term data reported by Spurgeon et al. showed that idelalisib given to patients with relapsed/refractory MCL resulted in an overall response rate of 40%, with higher rates in patients dosed at ≥100 mg BID [21]. Trial results of single-agent idelalisib in patients with indolent non-Hodgkin’s lymphoma (including FL, SLL, LPL/WM, MZL) showed an overall response rate (ORR) of 48% across all cohorts [63]. Among 11 patients with SLL, the response rate was 64%, whereas five of the 9 patients with LPL/WM responded, suggesting that idelalisib could be more effective in these subgroups [63].

Subsequently, a number of trials have examined idelalisib in combination regimens with a view to achieving clinically meaningful benefit. When idelalisib (I) was combined with rituximab (R) and/or bendamustine (B) in heavily-pretreated relapsed/refractory CLL patients, Coutre and coworkers documented an impressive response rates of 78, 82, and 87 percents for IR, IB, and IRB regimens respectively [83]. These combinations appear to be more effective than responses reported for RB (rituximab plus bendamustine) in previous studies of patients with relapsed/refractory CLL [84,85]. In the updated efficacy analysis of the current study, responses appear to be very durable [22]. The 2-year PFS and OS were 62% and 85% respectively [22]. Safety analysis indicated no overlap of key toxicities [22]. One study evaluated idelalisib plus ofatumumab as salvage therapy in relapsed/refractory CLL [86]. The study was small, evaluating only 20 patients, but interestingly, ORR was 94% in patients who had received 6 cycles or more, and appears to be superior to ofatumumab alone in this patient population [87]. The regimen was well tolerated and associated with marked and rapid reductions in lymphadenopathy within the first 2 cycles [86]. Given these favorable results, a phase III randomized, double-blind, placebo-controlled study has been initiated to assess the efficacy and safety of idelalisib in combination with bendamustine and rituximab versus placebo plus bendamustine and rituximab for previously treated CLL patients [88]. Likewise, another phase III randomized, controlled study is currently recruiting to examine idelalisib in combination with ofatumumab compared with ofatumumab alone in same patient population who had progressed after a purine analog and/or bendamustine [89].

In addition, a phase I trial employing the IR, IB, and IRB combination approaches was noteworthy for its associated response rates of 77%, 85%, and 79% respectively in patients with iNHL [90]. Though responses were high, it appears that they were not better than the 90% response rate achieved by the landmark study by Rummel et al. with rituximab and bendamustine in patients with relapsed/refractory iNHL [91]. Therefore, head-to-head comparison between idelalisib plus bendamustine and rituximab versus placebo plus bendamustine and rituximab in heavily-pretreated patients with iNHL has been initiated in a phase III trial [92]. At the same time, another phase III randomized trial will be comparing idelalisib plus rituximab versus placebo plus rituximab in similar patient population [93]. The primary endpoint of these studies is progression-free survival (PFS) [93].

The clear benefit of idelalisib in combination with chemotherapy and/or immunotherapy in CLL has lent support for the development of these approaches in patients with MCL. Preliminary results of a phase I study of 22 patients showed that the combinations of idelalisib and everolimus (IE), bortezomib (IV), or bendamustine plus rituximab (IRB) were active and tolerable in previously-treated patients with MCL [94]. Response rates were 25% for IE, 50% for IV, and 100% for IRB. Given that BR has been shown to elicit responses of 75 to 92 percent in similar patient population, the activity of IRB appears to be similar to what can be achieved with RB alone [91,95]. Nonetheless, these findings are preliminary and further research is required before any conclusions can be drawn.

The optimal first-line therapy for elderly patients with CLL is not currently known as most treatment options have not been directly compared. This remains the subject of multiple ongoing studies [19,96-98]. Based partly on the impressive response rate of idelalisib plus rituximab in the relapsed/refractory CLL setting [83], O’Brien et al. are addressing whether this IR regimen (R 375 mg/m^2^ weekly × 8 and idelalisib 150 mg bid continuously for 48 weeks) can be used in treatment-naïve, elderly patients with CLL/SLL [23]. Interim data regarding safety showed that the combination was tolerable, with diarrhea, pyrexia, chills, and fatigue being the most frequently reported adverse events. Of 48 patients evaluated for efficacy, the ORR was 96%, and estimated 24-month PFS is 91%, indicating that this approach is highly durable and paved the way for further study as upfront therapy in treatment-naïve elderly patients with CLL. Of note, six patients with del17p included in the study displayed 1 CR and 5 PR [23].

Overall, idelalisib looks impressive as both a single agent and when given in combination with standard therapies across multiple subtypes of non-Hodgkin’s lymphoma.

### Buparlisib (BKM 120, NVP-BKM120)

Buparlisib, also known as BKM 120 and NVP-BKM120, is an orally bioavailable, small molecule compound with potent, pan-class I PI3K inhibitory property against p110-α,-β, -δ, and -γ enzymes at IC50 of 52 nM, 166 nM, 116 nM, and 262 nM respectively [99]. As a derivative of pyridinamine, buparlisib shows great anti-proliferative activity in human gastric cancer cell lines, induces apoptotic cell death in multiple myeloma cells (ARP1, ARK, MM.1S, MM1.R and U266), and significantly reduces tumor volume and level of circulating human kappa light chain at 5 μM/kg/day in ARP1 SCID mouse model [99,100]. In vivo studies have also shown that buparlisib potently inhibits the growth of human xenografts models of metastatic brain melanoma, uterine endometriod carcinoma and carcinosarcoma, concomitant with suppression of PI3K phosphorylation [101,102]. Based on these promising preclinical data, buparlisib was advanced into clinical development.

The safety and preliminary clinical activity of buparlisib was first evaluated in a phase I study of 35 patients with advanced solid tumors by employing a dose-escalating design [103]. Overall, the compound was well tolerated. Dose limiting toxicities (DLTs) included grade 3/4 hyperglycemia, rash and mood alteration. The maximum tolerated dose (MTD) of 100 mg/day is deemed to be suitable for future studies. Aberrant PI3K signaling is common in glioblastoma multiforme (GBM) and confers worse prognosis [104], however buparlisib has demonstrated an ability to cross the blood–brain barrier in preclinical models. The preliminary results from two early phase trials of buparlisib in patients with relapsed/refractory GBM have been recently reported. Shih and colleagues found that buparlisib at 60 mg/day in combination with standard dose of bevacizumab was well tolerated [105]. Wen et al. showed that single-agent buparlisib at 100 mg/day is generally safe in patients with recurrent GBM. Major grade 3/4 toxicities were similar to those previously reported for the compound [106]. Buparlisib has also been evaluated in a number of other patient populations for which positive results have been reported. A combination of buparlisib and letrozole demonstrated activity at clinically relevant doses of each agent in hormone receptor (HR)–positive metastatic breast cancer (MBC) patients who had received prior aromatase-inhibitor therapy in a phase I study [107]. This potential superiority yielded by adding buparlisib to standard therapy in MBC has led to the initiation of two phase III trials. BELLE-2 and BELLE-3 are evaluating buparlisib with fulvestrant in postmenopausal women with HR+/HER2- advanced/ metastatic breast cancer after failure of aromatase inhibitor alone or aromatase inhibitor plus mTOR inhibitor treatment respectively [108]. A placebo-controlled phase II trial of buparlisib with paclitaxel in the first-line treatment of HER2-negative MBC (BELLE-4) is underway. A recent neoadjuvant phase II study of paclitaxel plus trastuzumab, with and without buparlisib (Neo-PHOEBE) in HER2-overexpressing breast cancer patients is also accruing. Though buparlisib in combination with geftinib was found to be safe, high frequency of severe late toxicities, including rash and diarrhea was noted in patients with EGFR TKI-resistant NSCLC in a phase IB study, and alternative dosing schedules are thus warranted in subsequent studies [109].

### GDC-0941

GDC-0941, a thienopyrimidine derivative, is another orally bioavailable, pan-class I PI3K inhibitor with equipotent activity (IC50 = 3 nM) against p110-α and -δ enzymes, and exhibits inhibitory action against p110-β and -γ at low nanomolar concentrations in kinase assays [110]. GDC-0941, as a single agent or in combination with other therapies, has demonstrated potent antitumor activity against a panel of mouse xenograft models of human glioblastoma, breast cancer, small bowel gastrointestinal stromal tumor (GIST), follicular cell lymphoma, liposarcoma, and NSCLC [110-116].

GDC-0941 is the first-in-human PI3K inhibitor to enter clinical trials. GDC-0941 monotherapy is generally well tolerated at doses below 450 mg once or twice a day in patients with advanced solid tumors [117]. The most common adverse events were nausea, diarrhea, vomiting, fatigue, decreased appetite, dysgeusia, and rash. In the updated efficacy analyses, clinically meaningful responses have been achieved with single-agent GDC-0941 in patients with endocervical carcinoma, breast cancer, soft tissue sarcoma, ovarian carcinoma, small bowel GIST and V600E mutant melanoma [117-120]. Given the single-agent activity of GDC-0941 in earlier studies, testing the drug in combination was seen as a logical step to maximize benefit. Concurrent administration of GDC-0941 and GDC-0973, a potent, selective, MEK1/2 inhibitor was well tolerated in patients with advanced solid tumors. No new safety signal has emerged, and clinical responses have been observed in patients with melanoma, pancreatic cancer, NSCLC, prostate cancer, and endometrioid cancer [121,122]. The synergistic efficacy of GDC-0941 and anti-VEGF directed therapy is being evaluated in a phase IB trial of GDC-0941 with paclitaxel and carboplatin, with and without bevacizumab in patients with advanced NSCLC. Partial responses were seen in 44% patients, including 1 pathologic CR upon resection of the primary lung lesion [123]. Phase II studies of GDC-0941 are underway.

### PX-866

PX-866 is a semisynthetic analogue of wortmannin with potent, irreversible, pan-class I PI3K inhibitory property against purified p110-α, -δ, and –γ enzymes at nanomolar concentrations in biochemical assays. Unlike wortmannin, PX-866 is a poor inhibitor of p110-β [124,125]. In preclinical studies, the compound alone or in combination with chemotherapy, radiation or other targeted cancer drugs, exhibited in vivo antitumor activity against numerous mouse xenograft models of human cancers [124,125].

Safety results from 52 patients indicated that PX-866 was well tolerated, with diarrhea being the DLT, and no drug-related serious hematologic adverse events reported [126]. The MTD of 8 mg was recommended for subsequent studies. Updated antitumor results of this trial demonstrated that PX-866 in combination with docetaxel was efficacious in patients with NSCLC and ovarian cancer (2 PR) [127]. Preliminary results from two randomized phase II clinical trials of PX-866 have been recently reported. In the first study, PX-866 displayed a very low ORR of 3% in 33 patients with recurrent GBM [128]. A second study explored the efficacy of PX-866 as second- or third-line treatment of docetaxel-naïve patients with recurrent or metastatic castration-resistant prostate cancer (CRPC). Of 16 patients evaluated for efficacy, no objective response was observed [129]. Other phase II trials are currently ongoing in a variety of tumor types.

### GDC-0032

GDC-0032 is a selective inhibitor of class I PI3K-α, - δ, and -γ isoforms in subnanomolar concentrations. It is an orally bioavailable small molecule with β isoform sparing inhibitory property. Treatment with GDC-0032 enhances activity of fulvestrant, resulting in tumor regressions and growth delay in preclinical animal models of human breast cancer [130,131]. A first-in-human phase IA clinical trial has been undertaken to assess the safety, pharmacokinetics and pharmacodynamics of GDC-0032 in 34 patients with locally advanced or metastatic solid tumors [132]. Results of this study indicated that the drug was well tolerated with hyperglycemia and fatigue being the dose-limiting toxicities. Five partial responses were observed in breast and NSCLC. Additional phase I trials are accruing patients.

### BAY 80–6946

BAY 80–6946 is a carboxamide derivative with potent antineoplastic activity characterized by reversible inhibition of p110-α and -β with IC50 of 0.469 nM and 3.72 nM respectively in biochemical assays, and growth-inhibitory effects in B-cell lymphoma and biliary tract carcinoma cell lines [133,134].

BAY 80–6946 was administered intravenously as 1-hour infusion once weekly for 3 weeks every month in a phase I dose-escalation trial of 17 patients with advanced solid tumors, including sarcoma, pancreatic, and esophageal cancers. It was well-tolerated [135,136]. Acute left ventricular dysfunction, liver dysfunction, renal insufficiency, hyperglycemia, and rash were the DLTs. The MTD was 0.8 mg/kg [136]. In a MTD expansion cohort study, 5 heavily pretreated patients demonstrated a PR to therapy [137]. More so, BAY 80–6946 has also demonstrated efficacy and safety among patients with both indolent and aggressive NHLs. These data have fuelled the enthusiasm for further clinical development of this compound either as a single agent or in combination regimens in patients with NHL [135].

### IPI-145

IPI-145 (formerly INK1197) is an oral, selective inhibitor of p110- δ and -γ isoforms at picomolar concentrations in enzyme assays. IPI-145 was initially developed as anti-inflammatory compound, displaying potent suppression of both B- and T-cell proliferation, and demonstrating dose-dependent anti-inflammatory effect in rat collagen induced arthritis (CIA) and adjuvant induced polyarthritis models.

The pharmacokinetics, safety and efficacy of IPI-145 have been studied in early phase clinical trials that included healthy subjects as well as patients with advanced hematologic malignancies [138,139]. The compound was well tolerated at doses up to 25 mg BID, exhibited excellent target inhibition (CD63 expression), and showed initial clinical activity in patients with iNHL, MCL, and CLL [139]. The main DLT was grade 4 neutropenia. Additional safety and efficacy data are expected from the ongoing trials.

### BEZ-235

BEZ-235 (formerly NVP-BEZ235), a novel imidazo-quinoline derivative, is a dual ATP-competitive PI3K and mTOR inhibitor with potent antagonist activity against p110-α, -β, -γ, -δ isoforms and mTOR (p70S6K) in nanomolar concentrations [140]. In vitro, BEZ-235 possesses strong anti-proliferative activity characterized by robust growth arrest in the G1 phase of many PTEN-negative malignancies, both in cell lines and in ex vivo cells [140,141]. Also BEZ-235 potently inhibits VEGF-induced cell proliferation and survival in vitro and VEGF-induced angiogenesis in vivo [142], and effectively reverses lapatinib-resistance in HER2 breast cancer cells [143]. Additionally, BEZ-235 as a single therapy or in combination with other agents exhibited antitumor activity against numerous mouse xenograft models of human cancers including gliomas, pancreatic cancer, sarcoma, ovarian cancer, renal cell carcinoma, breast cancer, and hepatocellular carcinoma [144-148].

The phase I study conducted by Arkenau et al. to determine the safety of single-agent BEZ-235 included 12 patients with advanced solid tumor with dose-level randomization into 4 cohorts [149]. Preliminary results of this study showed that BEZ-235 at 600 mg BID was well tolerated with mucositis being the most frequent DLT [149]. The combination of BEZ-235 and trastuzumab has been evaluated in a phase IB/II clinical trial in trastuzumab-resistant HER2+ MBC [150]. The doublet therapy demonstrated an acceptable safety profile and early sign of clinical activity. Preliminary safety data from another phase IB/II combination study of BEZ-235 with everolimus indicated that the regimen is safe, with no DLTs observed so far and the trial remains open to further accrual [151,152].

### BYL-719

BYL-719, a dicarboxamide analogue, is the first, orally bioavailable, potent selective inhibitor of PI3K-α with IC50 of 5 nM in kinase assays. Preclinical data suggested that the compound prevents phosphorylation of AKT and inhibits growth and PI3K signaling in breast cancer cell lines harboring *PIK3CA* mutations [153]. Dose-dependent antitumor activity was shown in a PIK3CA-mutant mouse xenograft models [153]. Treatment of MCF7 breast cancer cells and mouse xenograft models with BYL-719 and ganitumab, a fully human antibody against IGF1-R, resulted in synergistic, concentration-dependent growth arrest and tumor regression [154].

Based on these results, a phase I trial enrolled patients with *PIK3CA*-mutant advanced solid tumors, including estrogen receptor-positive (ER+) MBC [155]. Interim results showed that hyperglycemia, nausea, vomiting, and diarrhea were the DLTs, and 400 mg orally daily was declared as the MTD. Partial responses were seen in patients with breast, cervical, endometrial, ovarian, and head and neck cancer [155].

### BGT-226

BGT-226 (formerly NVP-BGT226) is another novel, dual pan-class I PI3K/mTOR antagonist with inhibitory property against p110-α, -β, and -γ isoforms with IC50 of 4 nM, 63 nM, and 38 nM in enzyme assays [156]. BGT-226 led to cell cycle arrest in the G0/G1 phase and inhibited growth in a variety of human cancer cell lines, including those that harbor the *PIK3CA* mutation [156-159]. Robust cancer cell death via apoptotic and non-apoptotic pathways, as well as induction of autophagy through microtubule-associated protein light chain 3B-II aggregation and p62 degradation are also associated with BGT-226 treatment [157]. In vivo studies have shown that oral doses of BGT-226 at 2.5 and 5 mg/kg for 3 weeks inhibit cytoplasmic expression of p70 S6 kinase and enhance autophagosome formation, translating into potent inhibition of tumor growth in human xenograft models [157].

A dose finding phase I study of BGT-226 indicated that the MTD was 125 mg per day or three times weekly, with 100 mg/day recommended as clinical dose for subsequent studies [156]. Most common BGT226-related adverse events included nausea, diarrhea, and vomiting. The best response of stable was demonstrated in patients with advanced solid tumors. The safety and efficacy data of other trials are awaited with great interest.

### PF-04691502

Like BGT-226, PF-04691502 is also a novel, ATP-competitive, dual pan-class I PI3K/mTOR inhibitor with activity against numerous human cancer cell lines at nanomolar concentrations [160,161]. PF-04691502 reduces levels of phosphorylated AKT (pAKT) T308 and S473, and its activity is not affected by presence of *PIK3CA* or PTEN mutations [160]. The compound also exhibits activity in animal models of KRAS-mutant non-small cell lung carcinoma xenografts, and thus potentially represents an effective therapeutic intervention for NSCLC patients with gefitinib- or erlotinib-resistant disease [160].

Updated data from the first-in-human phase I study aimed to establish the MTD, clinical activity, pharmacokinetics, and pharmacodynamics of PF-04691502 in 30 patients with advanced solid tumors. PF-04691502 appears to be safe and tolerable at a variety of dose levels [162]. Eight milligrams once daily is established as the MTD, and the most common adverse events noted were fatigue, nausea, vomiting, decreased appetite and rash. A phase II trial of PF-04691502 in combination with another dual PI3K/mTOR inhibitor, PF-05212384, in advanced endometrial cancer is currently recruiting.

### GDC-0980

GDC-0980 (formerly RG7422) is a novel, oral, dual PI3K/mTOR inhibitor synthesized using the GDC-0941 backbone [163]. In biochemical assays, GDC-0980 demonstrates its ability to inhibit the enzymatic activities of p110-α, -β, -δ, -γ and mTOR at IC50 of 5 nM, 27 nM, 7 nM, 14 nM, and 17 nM respectively [163]. In in-vitro experiments, potent anti-proliferative and pro-apoptotic effects of GDC-0980 were observed in prostate, breast and NSCLC cell lines, whereas modest activities were noted in pancreatic and melanoma cell lines [164]. In general, GDC-0980 demonstrated significant tumor growth inhibition in a wide range of xenografts derived from prostate, breast, ovarian, and lung cancer cell lines at doses of ≤7.5 mg/kg [163]. The compound was well tolerated and clinically efficacious in animal models at 55 mg given once daily without significant toxicities [165]. Recent preclinical studies have also shown that GDC-0980 combined with ABT888 (PARP inhibitor) and carboplatin seems to be approximately 2 times more potent than GDC-0980 alone at growth suppression in BRCA-competent triple negative breast cancer cell lines [166].

The safety, pharmacokinetics, pharmacodynamics and efficacy of GDC-0980 were first assessed in 33 patients with advanced solid malignancies in a dose-escalation phase I study [167]. Patients were enrolled in seven cohorts at dosage levels ranging from 2–70 mg once daily for 21 consecutive days of a 28-day cycle. Serious treatment-related adverse events included grade 3 maculopapular rash, symptomatic hyperglycemia, mucositis, and pneumonitis which resolved with drug cessation and medical management. Pharmacodynamic assessments revealed >90% inhibition of pAKT levels at dosage levels of 16 mg or above. GDC-0980 also showed promising antitumor activity, with RECIST and/or FDG-PET partial response rates up to 64% [167]. The recommended phase II dose for single-agent GDC-0980 is 40 mg daily. Several phase IB/II trials of GDC-0980 in combination with experimental or approved agents have been initiated. For example, the safety and efficacy of combination of GDC-0980 and abiraterone versus abiraterone alone are being evaluated in castration-resistant prostate cancer patients [168].

### GSK-2126458

GSK-2126458 is a potent, selective, second generation inhibitor of p110-α, -β, -γ, -δ, mTORC1, and mTORC2. It blocks PI3K/mTOR signaling at subnanomolar drug concentrations. Relative potency of GSK-2126458 in kinase assays is 100–1000 times greater than that of GDC-0980 [169]. Additionally, inhibition of the PI3K/mTOR pathway by this agent has shown activity in breast cancer cells in preclinical studies, particularly the *PIK3CA-*mutant subsets [169]. Dose-dependent antitumor activity was shown in BT474 mouse xenograft model, with significant response at a dose as low as 300 μg /kg.

While clinical experience with this compound is quite limited to date, the preliminary results of an early phase trial in seventy-eight patients with advanced solid tumors indicated that GSK-2126458 was safe, demonstrated on-target inhibition of PI3K, and diarrhea was the DLT [170]. Two patients with renal cell carcinoma and bladder cancer experienced partial response. When dosed once daily, a MTD of 2.5 mg was observed. Another phase I trial of GSK-2126458 in combination with oral MEK inhibitor GSK1120212 is planned.

### PF-05212384 (PKI-587)

Another novel, highly potent, dual PI3K/mTOR inhibitor is PF-05212384 (also known as PKI-587), which selectively binds to PI3K-α, PI3K-γ and mTOR and inhibits phosphorylation of both mTOR and AKT, and PI3K signaling. PF-05212384 leads to cell cycle inhibition and subsequent mitotic arrest, inhibition of proliferation, and apoptosis [171]. In vivo pharmacokinetics and pharmacodynamics suggested that intravenous PF-05212384 treatment is associated with low plasma clearance, high volume of distribution, long half-life, and robust antitumor efficacy in xenograft mouse models.

PF-05213384 is the first intravenously formulated PI3K/mTOR inhibitor to be tested in a clinical trial. In a phase I trial, Millham and colleagues used a modified continual reassessment method (CRM) for estimation of MTD. PF-05212384 was administered weekly at doses ranging from 10 mg to 319 mg [172]. A total of 47 patients with advanced or refractory solid tumors were enrolled, including 8 patients with colorectal cancer. DLTs included mucositis, rash, transaminase elevation, and hyperglycemia. The MTD was 154 mg weekly. No objective tumor response was observed, but 12 patients achieved stable disease during the study [172]. Recruitment to phase II trials is ongoing.

### XL765

A methylbenzamide derivative, XL765 (also known as SAR245409) is an orally active, multikinase (PI3K/mTOR) inhibitor with highly potent activity particularly for the p110-γ isoform in biochemical assays [173]. The compound was shown to inhibit proliferation and induce apoptosis in various tumor cell lines [173,174]. It demonstrated activity as monotherapy and in combination with temozolamide (TMZ) in GBM xenografts [175].

Data from a phase I dose escalation study of 34 patients with advanced or metastatic solid tumors indicate that XL765 is safe, and the most frequently observed adverse events included elevated liver enzymes, nausea and diarrhea [176]. XL765 combined with erlotinib demonstrated no additive toxicity, and generally well tolerated at daily doses up to 50 mg and 100 mg respectively [177]. Another trial showed that XL765 in combination with fixed standard dose of TMZ in 18 previously-treated patients with relapsed/refractory WHO grade III and IV astrocytic tumors was safe and generally well tolerated at doses up to 40 mg once daily [178]. Notably, the most serious treatment-related adverse events were rash, thrombocytopenia, and brain edema. Phase IB/II clinical trials of XL765 as a single agent and in combination with other targeted agents or cytotoxic chemotherapy are planned.

### XL147

XL147 (SAR245408) is an investigational methylbenzenesulfonamide derivative and a novel PI3K inhibitor. Preclinical studies demonstrated that XL147 exhibits pan-class I PI3K inhibitory property through reversible, competitive inhibition with ATP for p110-α, -δ, -γ, and -β enzymes at IC50 of 39 nM, 36 nM, 23 nM, and 383 nM respectively [179]. Additional preclinical data indicated that the main action of XL147 is inhibition of cell proliferation and growth, accompanied by abrogation of AKT and S6 phosphorylation, and reduction in cyclin D1 and pRB and an upregulation in levels of the CDK inhibitor p27 [179]. In a panel of HER2+ breast cancer cells, treatment with trastuzumab or lapatinib sensitizes tumor cells to the growth-inhibitory effect of XL147. Based on this preclinical rationale, XL147 has been evaluated in phase I and phase II clinical trials.

In an initial phase I trial with standard 3 + 3 dose-escalation design, 68 patients with advanced solid tumor were treated with XL147 administered on days 1–21 (21/7) every 4 weeks per treatment cycle or as a continuous daily dose (CDD) in 28-day cycle. The MTD, identified for both schedules, was 600 mg. Grade 3 rash was the DLT for the 21/7 schedule, whereas no DLTs were noted for the CDD dosing [180]. Pharmacokinetic data from another phase I study showed that treatment with XL147 plus erlotinib is associated with no major interaction, well-tolerated, and demonstrated robust concomitant EGFR and PI3K inhibition [181]. A clinical regimen of XL147, paclitaxel and carboplatin may synergistically augment suppression of PI3K signaling and enhance clinical effect. Interim data showed partial response rates of ≥ 42% by RECIST criteria in four patients with advanced solid tumor [182]. A recently presented study of patients with recurrent GBM has also provided further insight into the cellular pharmacodynamics and in vivo pharmacokinetics of XL147, where higher tumor to plasma drug concentration ratios were noted in resected tissue specimen, along with decreased Ki67 index consistent with inhibition of proliferation [183]. Additional clinical evaluation of this PI3K inhibitor is ongoing in phase I/ II studies.

## Conclusion and future directions

Phosphatidylinositol 3-kinases (PI3Ks) are attractive molecular targets for novel anti-cancer molecules. In the last few years, several classes of potent and selective small molecule PI3K inhibitors have been developed, and at least fifteen compounds have progressed into clinical trials as new anticancer drugs. Among these, idelalisib looks impressive as both a single agent and when given in combination with standard therapies across multiple subtypes of non-Hodgkin’s lymphoma. Phase III clinical trials are actively recruiting. Future trials of combining novel small molecule inhibitors against different signaling pathways as well as combination of these inhibitors with biological and biochemical agents may further enhance their clinical efficacy [41,184-189].

## Abbreviations

AMPK: 5′ adenosine monophosphate-activated protein kinase; BAD: Bcl-2-associated death promoter; FAK: Focal adhesion kinase; FOXO: Forkhead box protein O; GPCR: G protein coupled receptors; GSK3: Glycogen synthase kinase 3; JNK: c-Jun N-terminal kinases; LKB1: Liver kinase B1; MDM2: Mouse double minute 2 homolog; mTOR C1: Mammalian target of rapamycin complex 1; NF-κB: Nuclear factor kappa-light-chain-enhancer of activated B cells; PDK1: Pyruvate dehydrogenase lipoamide kinase isozyme 1; PI3K: Phosphatidylinositide 3-kinases; PIP3: Phosphatidylinositol (3,4,5)-triphosphate; PTEN: Phosphatase and tensin homolog; RHEB: Ras homolog enriched in brain; RTK: Receptor tyrosine kinase; SHIP: SH2-containing inositol phosphatase; TCS1/2: Two-component signal transduction protein 1/2.

## Competing of interests

The authors declare that they have no competing interests.
